# Autophagy: A Novel Horizon for Hair Cell Protection

**DOI:** 10.1155/2021/5511010

**Published:** 2021-06-29

**Authors:** Chang Liu, Zhiwei Zheng, Pengjun Wang, Shuangba He, Yingzi He

**Affiliations:** ^1^ENT Institute and Otorhinolaryngology Department, Eye and ENT Hospital, State Key Laboratory of Medical Neurobiology, Fudan University, Shanghai 200031, China; ^2^NHC Key Laboratory of Hearing Medicine (Fudan University), Shanghai 200031, China; ^3^Department of Otorhinolaryngology, Affiliated Sixth People's Hospital of Shanghai Jiaotong University, 600 Yishan Road, Shanghai 200233, China; ^4^Department of Otolaryngology Head and Neck, Nanjing Tongren Hospital, School of Medicine, Southeast University, Nanjing, ChinaChina

## Abstract

As a general sensory disorder, hearing loss was a major concern worldwide. Autophagy is a common cellular reaction to stress that degrades cytoplasmic waste through the lysosome pathway. Autophagy not only plays major roles in maintaining intracellular homeostasis but is also involved in the development and pathogenesis of many diseases. In the auditory system, several studies revealed the link between autophagy and hearing protection. In this review, we aimed to establish the correlation between autophagy and hair cells (HCs) from the aspects of ototoxic drugs, aging, and acoustic trauma and discussed whether autophagy could serve as a potential measure in the protection of HCs.

## 1. Introduction

As a general sensory disorder in human society, hearing loss was a major concern worldwide. The causes of hearing loss are infections, noise, aging, and ototoxic drugs. Autophagy is a common cellular reaction to stress that degrades cytoplasmic waste through the lysosome pathway. Autophagy not only plays major roles in maintaining intracellular homeostasis but is also involved in the development and pathogenesis of several diseases [1–6]. During a variety of pathological and physiological states, the activation of autophagy seems beneficial, as it removes damaged organelles or defends microbial infection and is important in various diseases related to metabolism or neurodegeneration [7].

Autophagy suppresses necroptosis and PARP-mediated cell death under cellular stress conditions. Early studies demonstrated that autophagy might be a prosurvival factor in many pathological processes [8]. In retinal ganglion cells, it was found that autophagy suppresses cell apoptosis and promotes cell survival, while the deletion of autophagy significantly decreases cell survival during the degeneration of the optic nerve [9]. In the auditory system, several studies revealed the link between autophagy and hearing protection and proved that the upregulation of autophagy might contribute to the alleviation of morphological damage in the inner ear [6, 10]. In this review, we aimed to establish the correlation between autophagy and hair cells (HCs), determined the effect of autophagy in hearing loss, and further discussed whether autophagy could serve as a potential measure in the protection of HCs.

## 2. Autophagy and Cochlear Development

Modulated by the growth factor signaling pathway, autophagy regulates cellular differentiation by providing energy and materials to the cells (such as immune cell [11]). Some studies proved the existence of autophagy in the inner ear of many species. For example, Atg4b^−/−^ null mice showed a defect in vestibular otoconia development, which might lead to the aberration of equilibrium [12]. Autophagy is involved in the neurogenesis in chicken's auditory spiral ganglion [13]. Moreover, HCs might present autophagic morphological features after ototoxic insults [14]. These data indicated that autophagy is crucial at the early stage of inner ear development. In the early postnatal stage of mice, auditory ribbon synapses are formed and matured with the development of cochlear HC stereocilia [15–18]. According to previous studies, although ATG5-deficient auditory HCs (including OHCs and IHCs) show well-functional morphology and mechanotransduction at P5, progressive accumulation of polyubiquitinated protein, as well as degeneration, is observed at P14 [19], which might eventually cause hearing loss. Furthermore, the treatment of postnatal mice with autophagy inhibitors before the onset stage of hearing induces long-term auditory disorders. The disruptions of autophagy have deleterious effects on the development of cochlear ribbon synapses in mice [20]. Together, these results strongly suggested that autophagy plays a critical role in the formation of HC morphology at the early postnatal stage.

## 3. Autophagy and Hearing Loss

Sensorineural hearing loss (SNHL) includes noise-induced hearing loss (NIHL), age-related hearing loss (ARHL), inherited hearing loss, and ototoxic drug-induced hearing loss (ODIHL). SNHL occurs because of the irreversible damage to hair cells (HCs) and spiral ganglion neurons (SGNs), both of which have very limited regeneration ability in adult mouse cochlea [21–26]. Thus, how to protect the cochlear HC is a key scientific question in the hearing research field.

### 3.1. Autophagy and Aminoglycoside-Induced Hearing Loss

As widely used clinical drugs, aminoglycosides (AGs) are famous for the broad antibacterial spectrum [27]. However, the application is limited because of the severe side-effects (including the ototoxicity), which might induce sensorineural hearing loss permanently and affect the life quality of patients significantly [28]. AGs cause damage to the outer hair cells (OHCs) of the basal turn, and with continuing drug exposure, damage spreads to the other turns as well as inner hair cells (IHCs) [29].

In the tissues with the need of high energy, such as HCs [30], phosphatase and tensin homolog- (PTEN-) induced putative kinase 1 (PINK1) is highly expressed [31]. According to a previous study, the suppression of PINK1 and the loss of HEI-OC1 cells were increased, with or without gentamicin (GM) exposure [32]. In addition, the PINK1 knockdown (KD) cells showed less expression of LC3B but a higher degree of p53 and activated caspase 3, indicating that PINK1 alleviates the GM-induced ototoxicity by inducing autophagy and resisting the level of p53 in HCs [32].

In the brain and neuroendocrine system, ubiquitin carboxyl-terminal hydrolase isozyme L1 (UCHL1) is highly expressed, which is important in maintaining synaptic structures, stabilizing ubiquitin, and regulating proteasomal or lysosomal degradation [33]. In the auditory system, UCHL1 was found to be downregulated in the cochlea by gentamicin (GM) in a time-dependent manner, and in damage or pathological condition, UCHL1 deficiency was found to be associated with the autophagy pathway [34, 35]. According to this study, UCHL1 was downregulated after GM treatment, and after GM exposure, siRNA or the pharmacological inhibitor (LDN-91946) treatment exacerbated the damage to the cochlear explants and HEI-OC1 cells [36]. The silencing of the UCHL1 protein blocked autophagic flux and the inhibition of LAMP1 and LC3 colocalization. These findings suggested that by downregulating UCHL1, GM might serve as a negative regulator to the autophagosome and lysosome fusion [36].

Fatty acids extracted from avocado oil have several functions, including promoting collagen synthesis, reducing inflammation, and wound healing [37]. In the brain tissue of diabetic rats, the seed oil attenuates oxidative stress and prevents mitochondrial dysfunction [38]. A recent study showed the protective effect of avocado oil on auditory HCs. Also, the avocado oil extract (DKB122) was shown to protect the HCs from neomycin-induced damage via upregulating antioxidant pathways, inhibiting inflammatory gene expression, and activating autophagy [39]. The level of p62 protein in HEI-OC1 cells is decreased in a dose-dependent manner after DKB122 treatment. Furthermore, p62 binds to LC3 and incorporates into the autophagosome; strikingly, the downregulation of intracellular p62 suggested that DKB122 upregulates the autophagy in HEI-OC1 cells.

### 3.2. Autophagy and Cisplatin-Induced Hearing Loss

Cisplatin is an anticancer drug used in clinical treatment. Based on the broad-spectrum chemotherapeutic effects, cisplatin is widely utilized in various cancers, such as testicular cancer, breast cancer, and head/neck cancer [40, 41]. However, progressive hearing loss could also be caused by high doses of cisplatin [6, 42–46]. Previous studies have shown that the accumulation of reactive oxygen species (ROS) after cisplatin treatment is a critical factor inducing HC damage [47–49].

m^6^A is a fat mass and obesity-associated (FTO) demethylase that regulates mRNA metabolism by catalyzing demethylation of m^6^A [50], while meclofenamic acid (MA), an anti-inflammatory drug, serves as a selective inhibitor [51]. A previous study reported that MA2 (a more active form of MA) treatment significantly reduces the apoptosis level of HEI-OC1 cells after cisplatin exposure. However, the effects of MA2 on cisplatin-induced HEI-OC1 death might not involve the participation of m^6^A, because no significant increase of m^6^A was observed after MA2 treatment [49]. The data showed that the level of autophagy was upregulated after cisplatin exposure, while after treatment of HEI-OC1 cells with MA2, the cisplatin-induced cell apoptosis and autophagy activation were significantly decreased [49]. These results suggested that the correlation between autophagy and cisplatin-induced apoptosis is complicated, and excessive autophagy might promote HC apoptosis.

As a Pou family transcription factor [52], Pou4f3 is necessary for HC differentiation, especially for the functional transduction and synaptic specialization [53, 54]. The mutation of Pou4f3 leads to the loss of HCs in the cochlea and subsequently results in severe hearing loss [55]. A recent study demonstrated that Pou4f3 participated in cisplatin-induced autophagy. After cisplatin treatment or AAV2-Pou4f3 shRNA transfection, Pou4f3 expression was decreased markedly. However, the levels of Beclin-1 and LC3-II were upregulated, indicating that Pou4f3 mutation promotes cisplatin-induced autophagy [56]. In addition, as the autophagy activator, rapamycin promoted HC apoptosis, while 3-MA inhibited HC apoptosis. This Pou4f3 mutation promotes HC apoptosis in the cochlea via autophagy [56].

A regulator of cell death, STAT1, was also reported to be involved in cisplatin-induced HC death [57, 58]. A previous study used siRNA to knockdown *STAT1* gene expression; consequently, the reduction of cisplatin-induced HC death was observed in vivo and in vitro [57]. In order to reveal the potential correlation between STAT1 and autophagy, other studies investigated the effect of STAT1 ablation on HC damage induced by cisplatin or GM. As shown in the study, comparing to the WT mice, both LC3-II conversion and Beclin-1 expression were increased in the explants from STAT1^−/−^ mice after cisplatin or GM treatment. However, 3-MA+cisplatin/GM treatment significantly reduced the level of surviving HCs in explants of STAT1^−/−^ mice, suggesting that autophagy is a potential protector in preventing cisplatin- and GM-induced HC death [59].

As a regulatory protein, glycogen synthase kinase 3*β* (GSK-3*β*) participates in a variety of physiological processes and the occurrence of several diseases [60–65]. Some studies also showed that as a downstream factor of AKT, GSK-3*β* is involved in the regulation of autophagy, while in the normal state GSK-3*β* suppresses the activation of autophagy. However, when abnormal external stimuli occur, the upstream kinase in the cells promotes the phosphorylation and leads to the inactivation of GSK-3*β* and blocks the inhibition on autophagy [66, 67]. In auditory research, gene knockout restored the inhibitory effect of GSK-3*β*, and the autophagy and cytoprotective effects in HEI-OC1 cells were enhanced successfully [68]. Furthermore, the GSK-3*β*-KO HEI-OC1 cells showed higher viability and higher autophagy rate after cisplatin treatment as compared to that of GSK-3*β*-WT HEI-OC1 cells. Moreover, cotreatment with 3-MA results in the reduction of autophagy accompanied by an upregulation of cell apoptosis [68]. These findings suggested that autophagy could be activated by downregulating the expression of GSK-3*β*, and autophagy exerts a protective effect against ototoxicity drugs.

As a member of the nucleotide-binding and oligomerization domain- (NOD-) like receptor (NLR) family, NLRX1 is important in regulating autophagy, ROS generation, and cell death in various kinds of cell types, responding to different stimuli [69–73]. In auditory research, it was found that NLRX1 was localized in the mitochondria of HEI-OC1 cells, and by activating the ROS/JNK signaling pathway and autophagy, NLRX1 sensitized the HEI-OC1 cells to cisplatin ototoxicity [74, 75]. According to the study, after treating HEI-OC1 cells with cisplatin, the increased expression of NLRX1 was synchronized with the activation of autophagy. However, by silencing the expression of NLRX1, the level of autophagy activation was reduced, and cell viability was increased [75]. Mechanistic studies revealed that by inhibiting ROS generation, autophagy activation and cell apoptosis could be successfully prevented in both HEI-OC1 cells and cochlear explants, providing a novel strategy against cisplatin-induced ototoxicity [75–78].

### 3.3. Autophagy and Noise-Induced Hearing Loss

Increased ROS production or decreased antioxidant activity causes oxidative damage, which is a key element causing noise-induced hearing loss (NIHL). However, ROS was also able to induce cellular defense processes, such as autophagy [79]. A recent study found that the level of oxidative stress in OHCs is noise-dose dependent and that the activation of autophagy exerts a protective effect against NIHL by alleviating the oxidative stress. As described previously, oxidative stress induced by temporary threshold shift (TTS) noise increases the level of LC3B in OHCs, and rapamycin treatment diminishes 4-HNE and 3-NT levels and decreases noise-induced HC loss. On the other hand, the reduction in LC3B via 3-MA or LC3B-siRNA increases the level of 3-NT and noise-induced cell death in OHCs [80].

Pejvakin, a peroxisome-associated protein from the gasdermin family, plays a critical role in sound-induced peroxisome proliferation [81]. Moreover, pejvakin participates in an early autophagic degradation of peroxisomes (pexophagy) in HCs after noise exposure [82]. According to the studies, pejvakin-mediated pexophagy prior to peroxisome proliferation protects the HCs against oxidative damage [83, 84]. As the lipid peroxidation in HCs was investigated through the assessment of the immunoreactivity of 4-HNE, an inverse correlation was established between pexophagy and 4-HNE, which suggested that pejvakin-mediated pexophagy plays a critical role in redox homeostasis and HC protection against noise-induced damage [82]. These results were also confirmed by the rescue experiments in Pjvk^−/−^ mice, as the viral transduction of Pjvk and LC3B cDNAs successfully restored the pexophagy and prevented the progress of oxidative stress [82]. Due to the similar clearance mechanisms owned by peroxisomes and mitochondria [85, 86], investigating whether mitochondrial autophagy has a protective effect against noise overexposure is essential.

Sleep deprivation (SD) also causes various inner ear diseases, such as hearing loss and tinnitus [87, 88]. The hypothalamic-pituitary-adrenal (HPA) axis is activated by successive SD, which subsequently leads to the upregulation of corticosterone [89], which in turn, activates the glucocorticoid receptors, resulting in the disruption of metabolic activity in the autophagy system [90]. The presence of corticosterone receptors in the auditory system has been demonstrated in many studies [91, 92], and it has been proved that high levels of corticosterone protect cochlear OHCs from various insults [93, 94]. Recently, the effects of SD on noise vulnerability were investigated with respect to the potential underlying mechanisms [95]. According to the study, the elevated expression of LC3B and autophagy activation is observed in the short-term SD+ acoustic trauma (AT) group, whereas repeated SD+AT group could not exert a similar effect. In addition, the SD+AT group exhibits high levels of corticosterone and severe OHC loss. This phenomenon could be attributed to the disruption of repeated SD to the homeostasis, resulting in the disorders of immunological malfunction [96, 97] and autophagy system disorder. However, the autophagy induced by short-term SD causes a protective effect on HCs in the apoptotic processes induced by oxidative stress. Thus, it is demonstrated that short-term SD exerts protective effects on HCs by regulating corticosterone and activating autophagy after noise exposure [95].

### 3.4. Age-Related Hearing Loss and Autophagy

As a predominant neurodegenerative disease, age-related hearing loss (AHL) is a common form of hearing loss [5, 98–102]. It is prevalent in adults aged ≥70 years, which results in social isolation, communication disorders, and a decline in physical function [103]. The SAMP8 strain is a classic model in studying the influence of aging on biological processes [104]. A previous study has revealed the mechanisms of ARHL, using SAMP8 mice, involved in the participation of oxidative stress, inflammation, and autophagic stress [105].

MicroRNAs (miRNAs) are endogenous, small, noncoding RNAs that control the stability of messenger RNA (mRNA). Recently, the existence of miRNAs in the cochlea has been proved, which suggested that miRNAs are critical in cochlear pathology [106, 107]. As a regulator controlling autophagy and cell death, miR-34a restrains autophagic flux by inhibiting autophagy protein ATG9a in HEI-OC1 cells [10, 108, 109]. Notably, the inhibitory effect of miR-34a to autophagy is mediated through multiple targets, such as SIRT1 and Bcl-2 [110–112]. As an NAD^+^-dependent histone deacetylase, SIRT1 is famous in the modulation of aging. The deacetylation of multiple autophagy-related proteins, including ATG7, ATG5, and ATG8 [113], or regulation of uncoupling protein 2 (UCP2) to stimulate mitophagy activates autophagy via SIRT1 [114]. According to a recent study, loss of HCs is observed in aged C57BL/6 mice, accompanied by the downregulation of autophagy and SIRT1 expression, and in HEI-OC1 cells, the decrease in SIRT1 leads to the reduction of LC3-II with an elevated level of p62. Moreover, silencing the expression of SIRT1 impaired the formation of the autophagosomes [115]. In addition, the long-term administration of resveratrol (SIRT1 activator) to the aging cochlea enhanced mitophagy, alleviated mitochondrial biogenesis, and improved the mitochondrial function [115]. Altogether, it suggested that the miR-34a/SIRT1 signaling pathway is a potential target for modulating autophagy, which is promising in delaying ARHL.

Inflammaging means a clinical condition in the elder with low-grade and chronic inflammation. Since it is related to several age-related diseases, it might increase the morbidity or mortality in the elderly. The long-term infection might alter the permeability and structure of the round window membrane [116], causing the permeation of lipopolysaccharide (LPS) into the inner ear [117]. When LPS enters the inner ear, it damages the HCs through multiple mechanisms, including inducing mitochondrial damage, accumulation of ROS, and activation of NF-*κ*B, SAPK/JNK, caspases, or other apoptotic pathways [118–121]. FoxG1 is a critical transcription factor belonging to the forkhead family. It regulates the proliferation and differentiation of cells, and the mutations in the *Foxg1* gene affect the development of axon and neuron [122]. During the development of the inner ear, FoxG1 is critical in maintaining the formation of HCs and the morphology of the cochlea [46]. FoxG1 also regulates the metabolism and biosynthesis of mitochondria [123–125], and hence, the unusual expression of FoxG1 results in opposing consequences to the function of mitochondria. Moreover, another study showed that the susceptibility of aging OC-1 cells to LPS-induced inflammation is regulated by FoxG1 via activated autophagy [5], thereby providing a potential target in AHL treatment.

### 3.5. Inherited Hearing Loss and Autophagy

The defection of mitochondrial tRNA in posttranscriptional modification is thought to be related to a variety of human hereditary diseases, including inherited hearing loss [126]. Recently, the molecular mechanism of deafness-related tRNAIle4295A>G mutations was deeply studied. It was found that abnormal tRNA metabolism may lead to mitochondrial translation disorders, respiratory deficiency, increased level of reactive oxygen species, and autophagy promotion [127]. However, whether the upregulation of autophagy in HCs is protective or not was not demonstrated in this study.

## 4. Other Factors Related to Autophagy in the Inner Ear

IGF-1 is a trophic factor belonging to the insulin family. It is involved in the development of the nervous system in adults, and hence, the mutations cause human syndromic deafness [128]. The correlation between IGF-1 and autophagy has been demonstrated in many studies. For example, activation of mTOR and IGF-1 downregulates autophagy in mammary epithelial cells [129]. On the other hand, in cultures of Purkinje neurons, IGF-1 promotes autophagy through the induction of autophagosome fusion with lysosomes [130]. However, in the auditory system, the role of IGF-1 in regulating autophagy does not seem crucial, as no significant difference was detected in the autophagic flux between IGF-1^+/+^ mice and IGF-1^−/−^ mice [131]; however, the role of IGF-1 in regulating autophagy should not be excluded out. Hearing impairment is also caused by cochlear ischemia [132–134]. Reportedly, the patients with carotid arterial sclerosis might suffer severer NIHL [135].

Hearing impairment can also be induced by cochlear ischemia [132–134]. Reportedly, carotid arterial sclerosis exacerbates the effects of noise exposure. Arterial sclerosis might be a contributor to hearing loss in the elderly, especially in those exposed to noise [135, 136]. According to a recent study, autophagy exerts a protective effect in ischemia-induced hearing loss, as the activation of autophagy occurs simultaneously with the recovery of hearing after reperfusion [137]. However, additional studies are required to understand the underlying mechanism.

## 5. Future Aspect

### 5.1. Using Autophagy as an Antioxidative Therapy

The role of autophagy in the auditory system is that of an antioxidant to protect HCs and hearing [6, 80, 138]. The accumulation of intracellular free radicals and ROS is increased by oxidative stress after injuries, and once the protective ability of the antioxidant system is surpassed, HC death happens [139, 140]. Previously, antioxidative therapies mainly focused on decreasing the ROS release or enhancing the antioxidant capacity. However, the efficiency of the conventional antioxidants is obviously limited to the already damaged organelles [141]. As autophagy can remove both oxidized proteins and impaired mitochondria at the same time, it offers a novel measure for the protection of HCs by eliminating the already oxidized proteins and organelles. Thus, autophagy can not only remove ROS accumulation but also eliminate injured mitochondria, suggesting that autophagy is an ideal candidate for antioxidation therapy [142]. Several studies have confirmed that autophagy is a basic antioxidant measure. After exposure of HEI-OC-1 cells to neomycin, the activation of autophagy (by rapamycin) enhances the expression of antioxidant genes, while the inhibition of autophagy exerts an opposite effect [6]. The upregulation of autophagy level suppresses the oxidative stress, thus exerting a protective effect on the cells. Hence, the appropriate elevation of autophagy might prevent SNHL induced by multiple factors.

### 5.2. Epigenetic and Autophagy

As epigenetic modifications participate in various biological processes, including transcriptional regulation and cell differentiation [143, 144], the dysregulation of epigenetic modifications may lead to developmental disorders, as well as cancer [145]. Although epigenetic research was under intensive focus in other biological systems, the understanding of epigenetics in ototoxic drug/noise/aged-induced hearing loss is not yet clear. In addition to MIR-34 described above, MIR-96 is also a promising target involved in autophagy and hearing loss treatment. As the first miRNA mutation associated with human deafness, the mutation in MIR-96 results in nonsyndromic SNHL [146]. Although miRNA is an essential factor in mRNA regulation, the downstream genes of miR96 in the cochlea are yet to be identified. Recent studies on the tumor [147] and brain injury [148] suggested that ATG7 could be modulated by miR-96. ATG7 activates ATG12 and ATG8 that are the essential components in the formation of autophagosomes [149]. According to a previous study, the reduced expression of miR-96 may directly increase the level of ATG7, thereby elevating the autophagosome synthesis and autophagy hyperactivation and eventually resulting in neuronal degeneration and death [150]. However, the correlation between ATG7 and miR-96 in the auditory system remains to be elucidated.

## 6. Conclusion

**Figure 1:**
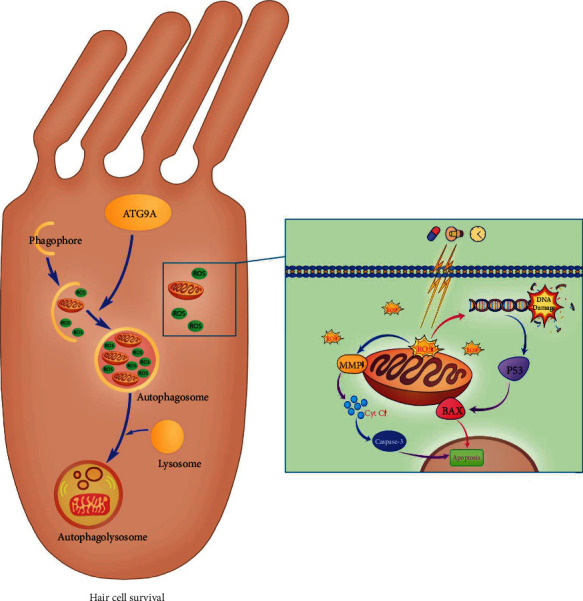
The correlation between autophagy and apoptosis in hair cell death.

Autophagy is involved in a variety of signaling pathways and plays a major role in the development and protection of HCs. Mitochondrion depolarization and ROS accumulation would happen when harmful stimulus exerted to HCs. If the damaged mitochondria cannot be cleared in time, the apoptosis pathway in HCs will be activated, leading to the death of HCs. However, with the activation of autophagy pathway, autophagosome could encapsulate the damaged mitochondria and fuse with lysosomes to form autophagosomes, thus degrading the damaged mitochondria and promoting the survival of HCs (Figure 1). However, autophagy seems to play a double-edged role in HC protection, some researchers believe that autophagy may aggravate HC damage, and others argue that autophagy can protect HCs from ototoxic factors, but the specific mechanism of autophagy is unclear. Along with the deepening of research, the mechanism of autophagy will be fully understood, and new treatments for hearing loss will be found in the future.
